# Kinetics of the soluble urokinase plasminogen activator receptor (suPAR) in cirrhosis

**DOI:** 10.1371/journal.pone.0220697

**Published:** 2019-08-29

**Authors:** Emilie Garnæs, Christian Mortensen, Lise Hobolth, Ove Andersen, Jan Nehlin, Søren Møller

**Affiliations:** 1 Center of Functional and Diagnostic Imaging and Research, Department of Clinical and Nuclear Medicine, Copenhagen University Hospital Hvidovre, Hvidovre, Denmark; 2 Gastro Unit, Medical Division, Copenhagen University Hospital Hvidovre, Hvidovre Denmark; 3 Clinical Research Center, Copenhagen University Hospital Hvidovre, Hvidovre, Denmark; Medizinische Fakultat der RWTH Aachen, GERMANY

## Abstract

**Background:**

The soluble urokinase plasminogen activator receptor (suPAR) is related to hepatic inflammation and fibrosis and has been suggested to participate in the development of liver cirrhosis. Therefore, the aim of the current study was to measure the concentration of suPAR in the hepatic vein of cirrhotic patients during a liver vein catheterization to identify a possible hepatic suPAR generation. Furthermore, we explored if suPAR levels were associated with the degree of cirrhosis and liver dysfunction.

**Methods and patients:**

We included 105 cirrhotic patients and 19 liver-healthy controls. Blood was sampled from the hepatic vein and the femoral artery and suPAR was measured by enzyme-linked immunosorbent assay.

**Results:**

We identified significantly higher median suPAR concentrations among the cirrhotic patients (7.2 ng/ml in the hepatic vein; 6.8 ng/ml in the femoral artery) compared to the controls (2.6 ng/ml, respectively, *p*-values <0.001). However, the median hepatic suPAR formation was 0.0 ng/ml in both groups. We observed significantly increasing suPAR levels according to higher Child classes (4.5 ng/ml, 6.9 ng/ml and 9.0 ng/ml, Child A, B, C respectively; *p*-value<0.001), and significantly higher median suPAR concentrations in patients with ascites versus patients without ascites (8.1 ng/ml versus 5.3 ng/ml, respectively, *p*-value<0.001). suPAR levels were significantly related to bilirubin (r = 0.48, *p*<0.001), the hepatic venous pressure gradient (r = 0.39, *p*<0.001), the cardiac index (r = 0.24, *p* = 0.02) and the plasma volume (r = 0.33, *p* = 0.001), whereas suPAR levels were significantly inversely related to albumin (r = -0.59, *p*<0.001), plasma coagulation factors (r-0.39, *p*<0.001), the mean arterial pressure (r = -0.28, p = 0.004), the systemic vascular resistance (r = 0.26, *p* = 0.007), the indocyanine green clearance (r = -0.51, *p*<0,001) and the galactose elimination capacity (r = -0.39, *p*<0.001).

**Conclusion:**

We identified elevated suPAR concentration in cirrhotic patients, which correlated significantly with the degree of cirrhosis and liver failure, but we were not able to demonstrate hepatic suPAR generation per se. This suggests that further investigations of the source of suPAR in cirrhotic patients need to be undertaken.

## Introduction

Liver cirrhosis is characterized by hepatic inflammation, fibrosis, and regeneration nodules, which lead to portal hypertension, and over time, progresses to the development of complications and a generalized organ dysfunction. It is the common end-stage of several chronic liver diseases, and it is associated with high morbidity and mortality with the only current definitive treatment option being liver transplantation.

Soluble urokinase plasminogen activator receptor (suPAR) is a part of the urokinase plasminogen activator/urokinase plasminogen activator receptor (uPA/uPAR) signal cascade, which has been suggested to play a key role in the development of liver cirrhosis [1,2]. uPAR is expressed on activated T-cells, neutrophils, macrophages, smooth muscle cells and endothelial cells [2] and upon activation by inflammatory stimulation uPAR is released in its soluble form suPAR. uPAR signaling orchestrates several immune functions such as cellular differentiation, migration, adhesion and invasion. Furthermore, activated uPAR can mobilize the serine protease plasminogen to its active form, plasmin, which is able to degrade fibrin. Increased suPAR levels is believed to reflect immune activation and several studies have reported elevated suPAR in inflammatory, infectious and cardiovascular diseases, and in cancer [3], linking heightened suPAR levels to worsened prognosis [4].

Similarly, increased suPAR levels have also been associated to hepatic inflammation and fibrosis in cirrhotic patients with exposure to both alcohol and hepatitis B and C [1,5,6]. Earlier studies have suggested that activated hepatic leucocytes are responsible for the suPAR generation in cirrhotic patients [7]; however, the only current data pointing directly towards a hepatic suPAR generation is a small study consisting of just 28 patients [8].

Liver vein catheterization is a safe and precise method of indirectly measuring the portal pressure [9–11], which is an important marker of prognosis and treatment response in cirrhotic patients.

Furthermore, blood sampling from the hepatic vein allows the estimation of a potential hepatic production of suPAR.

Therefore, the aims of the current study were to measure the levels of suPAR in the hepatic vein in a large group of cirrhotic patients, and to explore whether we could identify a hepatic suPAR generation. Furthermore, we investigated the association of suPAR with the degree of cirrhosis and organ dysfunction.

## Materials and methods

This study was conducted according to the Helsinki Declaration and approved by the Committee on health research ethics of the Capital Region of Copenhagen, Denmark (H-18045540). Furthermore, it was carried out according to the guidelines set by the Danish Data Protection Agency. Written and informed consent was obtained for all participants.

### Patient population

The study comprised 105 liver cirrhotic patients referred from the outpatient clinic at Hvidovre University Hospital during 2000–2013 for liver vein catheterization to determine the portal blood pressure. The diagnosis of cirrhosis was verified either by biopsy or based on established clinical, biochemical and ultrasonographic criteria. A summary of patient characteristics is presented in Table 1. Ninety-five patients had portal hypertension with an average hepatic venous pressure gradient of 15.4 mmHg. Forty-four of the patients had decompensated cirrhosis with ascites. Patients with previous or ongoing kidney disease were excluded from the study and none of the patients had experienced any episodes of hepatorenal syndrome.

**Table 1:** Patient characteristics.

					
	Child A	Child B	Child C	*p*-values	All cirrhotics
**Patient characteristics**					
**Gender (male/female)**	26/9	21/14	25/10		72/33
**Age (years)**	57.3 (53.7;61.0)	55.3 (51.3;59.2)	54.6 (51.3;57.9)	0.5	55.6 (53.7;57.8)
**Height (cm)**	172 (170;175)	169 (166;172)	175 (173;178)	0.006	172 (171;174)
**Body weight (kg)**	77.1 (72.3;81.8)	68.5 (62.4;74.6)	79.0 (72.0.85.9)	0.03	74.8 (71.4;78.3)
**BMI (kg/m2)**	25.9 (24.5;27.4)	23.7 (22.0;25.5)	25.5 (23.6;27.4)	0.15	25.1 (24.1;26.0)
**Body surface area (m2)**	1.90 (1.84;1.96)	1.79 (1.70;1.87)	1.93 (1.84;2.02)	0.02	1.87 (1.83;1.92)
**Ascites (-/+)**	35/0	18/17	8/27		61/44
**Alcohol-related cirrhosis/others**	17/18	24/11	25/10		66/39
**Blood chemistry**					
**S-albumin (g/l)**	40.9 (39.5;42.4)	33.2 (31.4;34.9)	24.8 (23.1;26.6)	<0.001	32.8 (31.8;34.6)
**S-creatinine (μmol/l)**	80.1 (75.9;84.3)	86.8 (81.8;91.8)	79.1 (72.1;93.8)	0.21	82.0 (78.8;85.2)
**S-bilirubin (μmol/l)**	10.7 (8.7;12.7)	19.9 (15.4;24.3)	57.8 (43.1;72.6)	<0.001	29.6 (23.1;36.1)
**S-alanine aminotransferase (U/l)**	40 (36;44)	42 (38;46)	51 (46;56)	0.33	44 (41;47)
**Plasma coagulation factors II. VII. X (units)**	0.64 (0.60;0.69)	0.57 (0.50;0.63)	0.40 (0.38;0.43)	<0.001	0.54 (0.51;0.57)
**B-platelets (E9/l)**	157 (142;172)	345 (242;449)	174 (146;201)	<0.001	199 (175;223)
**Hemodynamics**					
**Hepatic venous pressure gradient (mmHg)**	11.9 (9.9;13.8)	16.3 (14.5;18.1)	17.9 (16.4;19.3)	<0.001	15.4 (14.3;16.5)
Post-sinusoidal resistance (mmHg*min/l)	18.3 (6.1;30.5)	14.5 (11.4;17.6)	19.9 (13.7;26.0)	0.7	17.3 (12.4;22.2)
**Hepatic blood flow (l/min)**	1.15 (1.0;1.3)	1.4 (1.0;1.8)	1.2 (0.9;1.5)	0.5	1.2 (1.1;1.4)
**MAP (mmHg)**	99.2 (94.5;103.9)	93.7 (88.8;98.6)	89.8 (85.8;93.7)	0.01	94.3 (91.6;96.8)
**HR (min-1)**	72.8 (67.6;77.9)	73.8 (70.0;77.6)	78.6 (73.8;83.4)	0.15	75.0 (72.4;77.7)
**ICG clearance (ml/min)**	346.3 (280.1;412.5)	211 (176.6;246.0)	125.0 (94.4;155.6)	<0.001	233.7 (201.1;266.3)
**GEC (mmol/min)**	1.98 (1.81;2.16)	1.47 (1.35;1.60)	1.49 (1.34;1.64)	<0.001	1.66 (1.56;1.76)
**CI (l/min/m2)**	3.30 (3.04;3.56)	3.76 (3.29;4.23)	4.04 (3.68;4.39)	0.02	3.76 (3.55;3.96)
**Plasma volume (ml/kg)**	52.5 (49.6;55.4)	57.5 (54.5;60.4)	57.5 (54.0;61.0)	0.03	55.8 (53.9;57.6)
SVR (dyn*s/cm5)	1274 (1120;1427)	1090 (950;1229)	937 (824;1050)	0.002	1099 (1018;1181)

Data are presented as mean including 95% confidence intervals unless stated otherwise. Results are shown for all cirrhotic patients and subdivided into Child A, B, C groups. Column four with *p*-values describes a comparison of Child class A, B, and C groups.

Furthermore, we included 19 liver-healthy control patients who were admitted to the hospital under suspicion of mesenteric ischemia during 2008–2017. They were referred for measurement of splanchnic blood flow and mesenteric ischemia was not found.

### Liver vein catheterization, blood sampling and suPAR

All patients were without clinical signs of infections and had abstained from alcohol for at least one week before the liver vein catheterization. Diuretics and beta blockers were withdrawn 24 hours prior to the investigation, and the patients were fasting and had rested in supine position for at least one hour. None of the patients were treated with vasopressin analogues immediately prior to or during the study. The liver vein catheterization was done with catheterization of the femoral artery and the hepatic vein [12], and blood was sampled from these places using the catheters. Data on the hepatic venous pressure gradient (HVPG), the post-sinusoidal resistance, the hepatic blood flow (HBF), the mean arterial pressure (MAP), the indocyanine green (ICG) clearance, the galactose elimination capacity (GEC), the cardiac index (CI), the plasma volume and the systemic vascular resistance (SVR) was obtained as previously described [12].

The splanchnic flow measurement was performed similarly with catheterization of the femoral artery and the hepatic vein, and blood was also sampled from there.

Plasma was separated by centrifugation and stored at -80° Celsius. suPAR concentration was determined by enzyme-linked immunosorbent assay (ELISA) according to manufacturer’s instruction (suPARnostic, Virogates, Denmark) and hepatic suPAR formation was calculated as the concentration of suPAR in the hepatic vein minus the concentration of suPAR in the femoral artery [13].

### Statistical analysis

Statistical analyses were done in SPSS (IBM, New York, USA). To compare the means of the clinical, biochemical and hemodynamic characteristics in each Child class, one-way ANOVA analyses were done. Data on suPAR levels were skewed why they are presented as medians including maximum and minimum values. To compare suPAR levels in cirrhosis patients versus liver-healthy patient controls, we performed a Mann-Whitney test. To compare suPAR levels in the Child A, Child B and Child C groups, a Kruskal Wallis test was done. To calculate correlations between suPAR and different markers, we calculated Spearman correlations presented as the coefficient r and the following *p*-value. *p*-values <0.05 were considered significant.

## Results

### Patient characteristics

The patient characteristics are presented in Table 1. Among the cirrhotic patients, we observed a higher frequency of men compared to women, and the mean age was 56 years. In comparison, the liver-healthy patient controls were older with a mean age of 70 and a male:female ratio of 8:11 (data not shown). A history of alcohol abuse was defined as an alcohol consumption exceeding 50 g/day for more than 5 years. During the study all patients had abstained from alcohol for at least 1 week before the investigations and had no signs of withdrawal symptoms at the time of the study.

Approximately 2/3 of the cirrhotic patients had a history of alcohol abuse whereas the rest had liver cirrhosis secondary to other causes. Furthermore, 2/3 of the cirrhotic patients presented with ascites at the time of the liver vein catheterization.

Looking at the hemodynamics, a higher HVPG was evidenced with increasing Child score (Table 1). Furthermore, we observed a lower MAP and SVR, and a higher heart rate (HR), CI and plasma volume in the Child C group compared to the Child A group. Finally, the liver function measured by the GEC and the ICG clearance decreased correspondingly with a higher Child score (Table 1).

### suPAR in cirrhotic patients and controls

We identified a median suPAR concentration of 7.2 ng/ml in the hepatic vein and 6.8 ng/ml in the femoral artery of cirrhotic patients. This was significantly higher than the suPAR levels of the liver-healthy controls (*p*-values <0.001, respectively, Table 2). However, the median hepatic suPAR formation was 0.0 ng/ml both in the cirrhotic patients and in the liver-healthy controls.

**Table 2:** Hepatic suPAR formation.

	Cirrhosis (n = 105)	Controls (n = 19)	*p*-value*	Child A (n = 35)	Child B (n = 35)	Child C (n = 35)	*p*-value**
**suPAR concentration**	Median (min/max)	Median (min/max)		Median (min/max)	Median (min/max)	Median (min/max)	
**The femoral artery (ng/ml)**	6.8 (1/29.4)	2.6 (1.3/7.8)	<0.001	4.5 (1.0/17.8)	6.9 (3.2/16.3)	9.0 (4.8/29.4)	<0.001
**The hepatic vein (ng/ml)**	7.2 (1/27.4)	2.6 (1.3/7.8)	<0.001	4.4 (1.0/17.6)	7.4 (3.0/16.1)	8.8 (4.8/27.4)	<0.001
**Hepatic suPAR generation (ng/ml)**	0.0 (-2.6/8)	0.0 (-1.9/1.2)	0.3	0.1 (-2.1/3.1)	0.0 (-1.2/1.7)	0.0 (-2.6/8)	0.4

Data are presented as median including minimum and maximum value. The first column of p-values (*) is a comparison of suPAR in cirrhosis versus controls. The second column of p-values (**) is a comparison of suPAR in the Child A, Child B, and Child C groups.

After subdivision of cirrhotic patients into Child A, B and C classes, we found significantly different suPAR concentrations in each Child class, with increasing suPAR levels according to higher Child classes (*p*-value<0.001, Table 2). However, the median hepatic suPAR generation did not differ according to each Child class (0.1 ng/ml, 0.0 ng/ml and 0.0 ng/ml in Child A, Child B and Child C classes, respectively).

**Fig 1:**
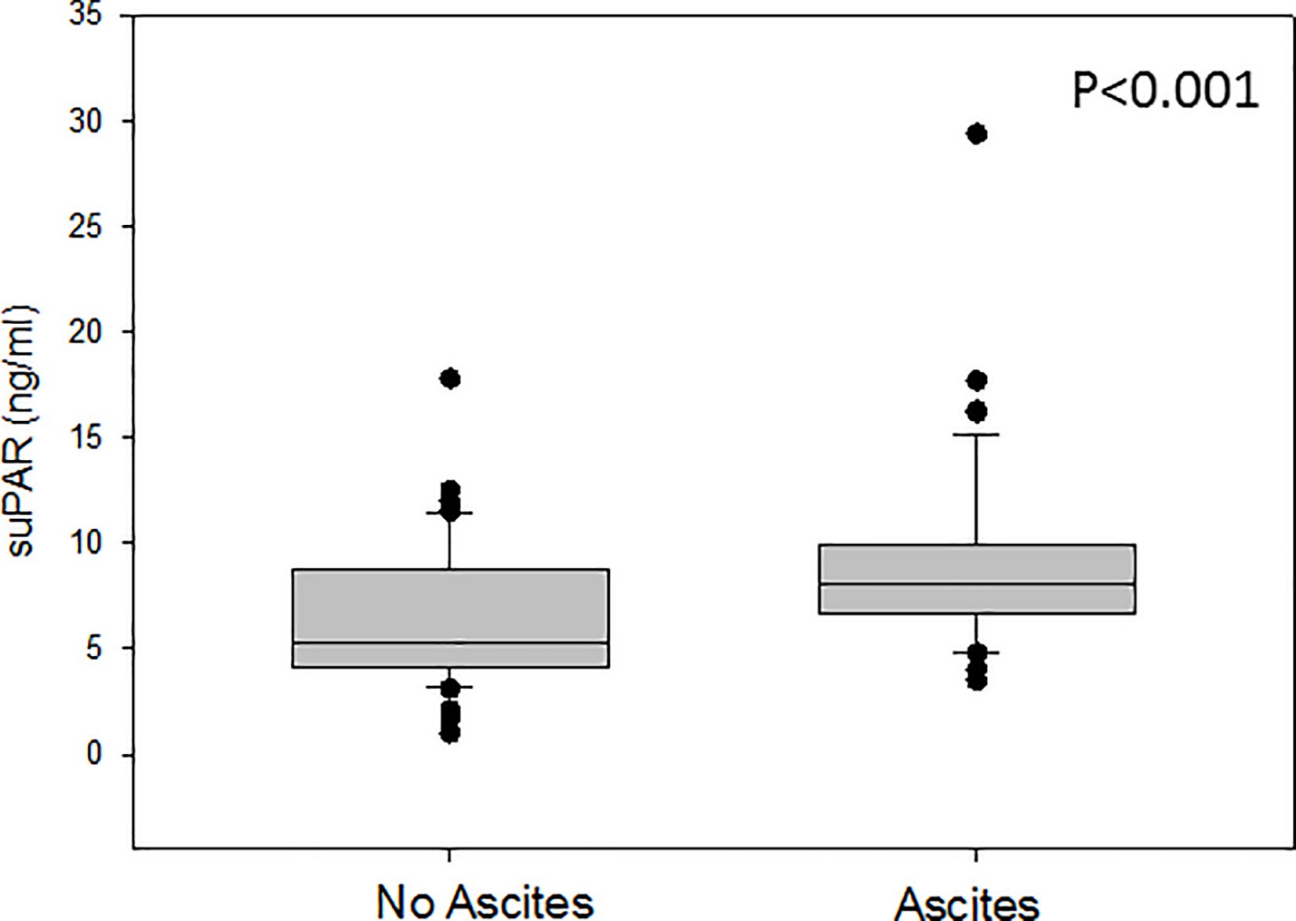
suPAR concentrations in the femoral artery according to the presence of ascites versus no ascites. There was a significantly higher suPAR concentration in the femoral artery among patients with ascites (8.1 ng/ml) versus patients without ascites (5.3 ng/ml, p<0.001).

We also observed a significantly higher median suPAR concentration in the femoral artery of patients with ascites versus patients without ascites (5.3 ng/ml vs. 8.1 ng/ml, respectively, *p*-value<0.001, Fig 1). The same was true for the median suPAR concentration in the hepatic vein of patients with and without ascites (data not shown). However, there was no difference in the suPAR concentrations of the hepatic vein and the femoral artery among patients with and without ascites (data not shown).

### Correlation between suPAR and biochemical and hemodynamic markers

The concentration of suPAR in the femoral artery was significantly correlated to bilirubin (r = 0.48, *p*<0.001), HVPG (r = 0.39, *p*<0.001), CI (r = 0.24, *p* = 0.02) and plasma volume (r = 0.33, *p* = 0.001). Whereas, there was a significant inverse relation between suPAR levels in the femoral artery and albumin (r = -0.59, *p*<0.001), plasma coagulation factors (r-0.39, *p*<0.001), MAP (r = -0.28, p = 0.004), ICG clearance (r-0.51, *p*<0.001), GEC (r = -0.39, *p*<0.001) and SVR (r = 0.26, *p* = 0.007) (Table 3), but suPAR did not correlate significantly with alanine aminotransferase as an indicator of hepatocellular damage (r = 0.17, p = 0.09).

**Table 3:** The correlation between suPAR levels in the femoral artery and biochemical and hemodynamic markers.

Correlation to suPAR femoral artery	Spearman correlation	
	r	*p*-value
**Blood biochemistry**		
**Albumin**	-0.59	<0.001
**Bilirubin**	0.48	<0.001
**Plasma coagulation factors II. VII. X**	-0.39	<0.001
**Hemodynamics**		
**Hepatic venous pressure gradient**	0.39	<0.001
**Post-sinusoidal resistance**	0.2	0.07
Hepatic blood flow	0.04	0.7
**MAP**	-0.28	0.004
**Heart rate**	0.18	0.06
**Cardiac index**	0.24	0.02
**Plasma volume**	0.33	0.001
**Systemic vascular resistance**	-0.26	0.007
**Liver function**		
**ICG clearance**	-0.51	<0.001
**Galactose elimination capacity**	-0.39	<0.001

Data are presented as the Spearman correlation coefficient r and *p*-value.

In the total patient population, 8 patients had a serum creatinine level that exceeded 130**μ**mol/l. Circulating suPAR correlated significantly with serum creatinine (r = 0.25, p<0.01). The median suPAR concentration was 9.4 (6.7–17.8) in patients with a serum creatinine above 130 **μ**mol/l compared to 6.7 (1.0–29.4) in those patients with a serum creatinine below this level (p<0.01).

Furthermore, there was a tendency towards a relation between the concentration of suPAR in the femoral artery and post-sinusoidal resistance (r = 0.2, *p* = 0.07) and HR (r = 0.18, *p* = 0.06) (Table 3).

## Discussion

In this study of 105 liver-stable cirrhotic patients without infection, we demonstrated a high suPAR concentration in both the hepatic vein and in the femoral artery compared to liver-healthy patient controls. To the best of our knowledge this has not been demonstrated previously. suPAR concentrations increased significantly with higher Child class, and suPAR concentrations were significantly higher among patients with ascites versus patients without ascites, which correlated with hepatic vein hypertension. Furthermore, we observed significant correlations between suPAR levels and liver biochemistry, liver function tests and both liver and systemic hemodynamics indicating a close relationship between suPAR concentration and the severity of the liver cirrhosis and its organ dysfunction.

In this large study, we found no sign of hepatic suPAR generation. Our results are in line with previous studies demonstrating increasing suPAR levels according to the stage of fibrosis and inflammation [1,6,7,14,15]. These characteristics are well described in the pathogenesis of liver cirrhosis, where chronic exposure to toxic agents such as alcohol or liver viruses causes liver injury, which activate the hepatic stellate cells into depositing extracellular matrix including fibrin [16] leading to liver inflammation. uPA has been suggested to be important in liver repair mechanisms because it contributes to fibrinolysis, extracellular matrix degradation and immune modulation [17]; however, the exact role of the uPA/uPAR system in liver cirrhosis remains ambiguous. In experimental mouse models, uPA- and uPAR knockout mice showed decreased hepatic fibrosis [17]; whereas, other mice studies showed that abrogation of the uPA/uPAR interaction increased fibrin deposition and fibrin associated inflammation [18] and suggested that uPA/uPAR signaling supported liver repair [19]. A link in this may be suPAR; suPAR has been proposed to negatively regulate uPA/uPAR signaling by acting as an uPA scavenger [20] and thereby inhibiting fibrinolysis [21]. Furthermore, it has been demonstrated that suPAR fragment_DII-III_ can act as a chemo-attractant for neutrophils and monocytes [22], which can lead to further suPAR release, progressing to a vicious circle of inflammation. From a clinical point of view it would be relevant to relate suPAR levels to the histological stage of fibrosis as assessed by liver biopsy, elastometry, or serological markers of fibrosis. These relations are important topics for future research. Since hepatocellular damage may lead to increased fibrogenesis and inflammation an association between suPAR and markers of hepatocyte damage such as increased alanine aminotransferase could be expected. However, we were unable to demonstrate such a relation in the present patient population, which may attributed the chronic nature of the disease in this relatively stable patient population.

Unexpectedly, we were not able to demonstrate a hepatic suPAR formation in this cohort of clinical stable cirrhotic patients without evidence of the presence of bacterial pathogens. However, a possible intra-hepatic generation of suPAR in the presence of bacterial pathogens generating an immune response with hepatic synthesis of Toll-like receptors inducing a hepatic increase in suPAR, cannot entirely be ruled out in this study.

New evidence has demonstrated a causative role for circulating suPAR in focal segmental glomeruloscleroses, where bone marrow derived immature myeloid cells seems to be the main source of the suPAR [23,24]. This suggests a functional connection between the bone marrow and the kidney; whereas our data also suggest a systemic suPAR increase. Interestingly, one the sequela in liver cirrhosis is kidney failure; still, it is currently unknown whether elevated suPAR also leads to decreased kidney function in cirrhotic patients. Others have suggested that impaired renal or biliary clearance might cause the increased suPAR levels in cirrhotic patients [25]. In patients with acute liver failure, suPAR production was associated with distinct immune cell intra-hepatic accumulation and strong up-regulation of intra-hepatic uPAR mRNA. The finding that suPAR correlated with serum creatinine and were particular higher in the 8 patients with higher serum creatinine above 130 **μ**mole/l points to the assumption that suPAR is also a marker of progress of complications to portal hypertension such as hepatic nephropathy.

In conclusion, we identified an elevated suPAR concentration in non-infected patients with cirrhosis, which correlated significantly with disease stage, liver function and hemodynamic consequences, but we did not demonstrate a hepatic suPAR formation. Our findings suggest that suPAR is involved in the inflammatory process leading to hepatic dysfunction; however, the primary origin of suPAR generation is located outside the liver tissue per se. This must lead to further research to investigate the source of suPAR in patients with liver cirrhosis.

## Supporting information

S1 Dataset Minimal dataset. (PDF)

## Abbreviations

BMI: Body mass index
CI: Cardiac index
GEC: galactose elimination capacity
HR: Heart rate
HVPG: hepatic venous pressure gradient
ISG: interferon-stimulated gene
MAP: mean arterial pressure
suPAR: Soluble urokinase plasminogen activator receptor
SVR: sustained virologic response
uPA: urokinase plasminogen activator
uPAR: urokinase plasminogen activator receptor
