# Supplementary material for: Kinetics of the soluble urokinase plasminogen activator receptor (suPAR) in cirrhosis
Source: PLoS One. 2019 Aug 29;14(8):e0220697. doi: 10.1371/journal.pone.0220697 (PMC6715219; doi:10.1371/journal.pone.0220697)
Supplement: S1 Dataset — (PDF) [file pone.0220697.s001.pdf]

ID no	Diagnosis	Child	suPAR arteria	suPAR vena hepatica	Difference
3013	alc.cirr.	A	4,3	4,1	-0,2
2765		A	4,5	4,7	0,2
365		A	4,3	4,1	-0,2
1961		A	6,2	6,1	-0,1
1786		A	3,8	4,0	0,2
1986		A	5,5	4,9	-0,6
491		A	3,2	3,8	0,6
2001		A	4,9	4,8	-0,1
375		A	4,5	4,4	-0,1
705		A	9,4	9,4	0,0
323		A	5,6	6,8	1,2
745		A	5,0	2,9	-2,1
2051		A	3,4	3,5	0,1
993		A	5,1	5,2	0,1
1502		A	11,2	11,9	0,7
2297		A	4,3	4,3	0,0
2725		A	3,1	4,2	1,1
283		A	4,3	4,7	0,4
1610	Autoimmu	A	2,1	2,0	-0,1
2080	Budd Chiar	A	4,1	4,0	-0,1
462	cir.hep. Ex.	A	4,2	4,4	0,2
2848		A	4,6	4,8	0,2
997		A	1,0	1,0	0,0
2533		A	3,2	3,3	0,1
1763		A	10,8	11,5	0,7
2129		A	11,5	11,8	0,3
2759		A	3,1	3,2	0,1
733		A	17,8	17,6	-0,2
2127	cirr.hep.ex.	A	6,4	7,4	1,0
2516		A	3,5	3,8	0,3
3065		A	1,7	1,7	0,0
2652		A	4,1	4,1	0,0
1823		A	5,8	5,7	-0,1
1811	Water mel	A	12,5	12,6	0,1
1193		A	5,8	8,9	3,1
955		B	5,3	5,3	0,0
313	alc.cirr.	B	5,2	6,6	1,4
1441	alc.cirr.	B	8,7	8,7	0,0
955	alc.cirr.	B	9,4	9,4	0,0
2440	alc.cirr.	B	9,7	9,4	-0,3
2393	Alc.cirr.	B	8,3	8,6	0,3
2684		B	9,2	9,7	0,5
296	Alc.cirr.	B	5,9	5,5	-0,4
2342	alc.cirr.	B	8,1	7,9	-0,2
376	Alc.cirr.	B	8,6	8,8	0,2
961	alc.cirr.	B	6,7	7,7	1,0
163	Alc.cirr.	B	5,1	5,5	0,4
284	alc.cirr.	B	7,4	7,4	0,0
351	alc.cirr.	B	9,1	7,9	-1,2

955		B	3,2	3,4	0,2
743		B	4,6	4,2	-0,4
2455	Ascites	B	3,5	3,8	0,3
927	cir.alc.o.p.	B	7,9	7,6	-0,3
1110	cir.ex.alc.	B	7,4	7,6	0,2
1913	cir.ex.alc.	B	5,6	5,8	0,2
882	cir.hep.alc	B	8,8	8,3	-0,5
311	cir.hep.alc	B	8,5	8,7	0,2
1905	cir.hep.alc	B	4,9	5,5	0,6
785	cir.hep.alc	B	4,0	3,6	-0,4
436	Cir.hep.alc	B	16,3	16,1	-0,2
847	cir.hep.alc	B	6,7	6,5	-0,2
197	cir.hep.alc.	B	9,3	9,5	0,2
388	cirr.hep.	B	6,7	6,3	-0,4
241	cirr.hep.ex.	B	6,9	6,9	0,0
3335	cirr-CAH-C	B	3,3	3,0	-0,3
949	Hep.cel.car	B	3,7	4,0	0,3
1428	Kronisk aut	B	3,5	3,3	-0,2
922	krypt.cirr.	B	3,6	3,2	-0,4
378	post.hep.ci	B	8,7	8,2	-0,5
1030		B	8,2	9,9	1,7
886		C	11,5	11,5	0,0
614		C	6,4	7,2	0,8
208		C	29,4	27,4	-2,0
295		C	12,0	15,9	3,9
2442		C	8,1	7,0	-1,1
239		C	6,8	6,9	0,1
101		C	13,8	13,7	-0,1
2559		C	7,0	6,8	-0,2
1109		C	9,6	10,1	0,5
267		C	16,2	13,6	-2,6
2394		C	7,0	8,2	1,2
793	alc.cirr.	C	8,1	7,6	-0,5
1169	Alc.cirr.	C	6,3	6,2	-0,1
1989	alc.cirr.	C	6,1	5,1	-1,0
2629	Alfa-1-antit	C	9,0	8,8	-0,2
449		C	6,8	7,2	0,4
397		C	8,0	8,1	0,1
623		C	4,8	5,1	0,3
212		C	10,7	10,7	0,0
399		C	14,1	14,0	-0,1
300		C	10,0	9,2	-0,8
1024		C	11,1	12,1	1,0
2449	cir.hep.alc	C	9,1	10,1	1,0
1161	cir.hep.alc	C	11,6	11,5	-0,1
185	cir.hep.alc.	C	11,5	11,2	-0,3
1097		C	5,2	4,8	-0,4
595		C	7,7	7,6	-0,1
2340		C	17,7	17,9	0,2
1961		C	7,9	8,4	0,5

2168	C	6,6	6,4	-0,2
3419	C	7,9	8,0	0,1
217	C	10,0	9,7	-0,3
2169	C	12,0	12,0	0,0
2357	C	10,0	18,0	8,0
2467	C	7,2	7,4	0,2

ID no.	Diagnosis	Age	Body high	Ideal body	Body weig
3013	alc.cirr.	55	177,5000	70,6250	67,0000
2765	alk.cirr	62,0	168,0	63,50	88
365	alk.cirr	64,0	172,0	66,50	68
1961	Alk.cirr.	63,0	168,0	63,50	74
1786	Alk.cirr.	57,0	160,0	57,50	70
1986	Alk.cirr.	57,0	160,0	57,50	68
491	Alk.cirr.	68,0	171,0	65,75	73
2001	Alk.cirr.	52,0	180,0	72,50	92,4
375	Alk.cirr.	66,0	178,0	71,00	81,5
705	Alk.cirr.	64,0	182,0	74,00	78
323	Alk.cirr.	49,0	185,0	76,25	70
745	Alk.cirr.	71,0	182,0	74,00	104
2051	Alk.cirr.	57,0	170,0	65,00	63
993	Alk.cirr.	54,0	175,0	68,75	96
1502	Alk.cirr.	61,0	158,0	56,00	81
2297	Alk.cirr.	52,0	177,0	70,25	74,7
2725	Alk.cirr.	63,0	175,0	68,75	68
283	Alk.cirr.	56,0	179,0	71,75	78,3
1610	Autoimmun hepatitis	19	174	68,00	74
2080	Budd Chiari o.p.	48	158,00	56,0000	70,3000
462	cir.hep. Ex. Alc o.p.	73	172	66,50	92,0000
2848	Cirr.ex.alc.	60,0	162,5	59,38	64,9
997	Cirr.ex.alc.	39,0	171,0	65,75	71
2533	Cirr.ex.alc.	45,0	183,0	74,75	111
1763	Cirr.ex.alc.	55,0	177,0	70,25	93,3
2129	Cirr.ex.alc.	49,0	193,5	82,63	50
2759	Cirr.ex.alc.	61,0	170,0	65,00	86,6
733	Cirr.ex.alc.	65,0	178,0	71,00	79
2127	cirr.hep.ex.alc	53	172,0000	66,5000	87,0000
2516	Hep.C	51,0	165,0	61,25	70
3065	Miltvenetrombose o.p.	59,0	175,0	68,75	88,9
2652	PBC	52	161	58,25	60
1823	PH o.p./Hep.carc.	67,0	163,5	60,13	50
1811	Water melon stomack	81	170	65,00	64
1193		59	173	67,25	90
955	Alc.cirr.	54	182	74,00	79
313	alc.cirr.	46	178,00	71,00	80,0000
1441	alc.cirr.	55	174,00	68,0000	56,4000
955	alc.cirr.	57	169,0000	64,2500	76,0000
2440	alc.cirr.	34	157,0000	55,2500	51,5000
2393	Alc.cirr.	53	170	65,00	74
2684	Alc.cirr.	65	158	56,00	53
296	Alc.cirr.	65	160	57,50	41
2342	alc.cirr.	53	165,00	61,2500	52,5000
376	Alc.cirr.	38	168	63,50	61,9
961	alc.cirr.	50	172,0000	66,5000	66,0000
163	Alc.cirr.	45	180	72,50	72,4
284	alc.cirr.	46	160,00	57,5000	42,0000
351	alc.cirr.	57	165,00	61,2500	63,6000

955	Alk.cirr.	54,0	184,0	75,50	83
743	Alk.cirr.	63,0	163,0	59,75	82
2455	Ascites	53	175,5	69,13	94
927	cir.alc.o.p.	58	178	70,63	104,0000
1110	cir.ex.alc.	58	162	59,00	69,6000
1913	cir.ex.alc.	44	178	71,00	105,0000
882	cir.hep.alc	58	164	60,13	52,4000
311	cir.hep.alc	55	173	67,25	68,0000
1905	cir.hep.alc	75	165	61,25	81,0000
785	cir.hep.alc	67	175	68,75	92,1000
436	Cir.hep.alc	52	160	57,13	31,6000
847	cir.hep.alc	56	173	67,25	60,0000
197	cir.hep.alc.	55	169	64,25	86,0000
388	cirr.hep.	68	162,0000	59,0000	52,0000
241	cirr.hep.ex.alc	55	174,0000	68,0000	79,0000
3335	cirr-CAH-C	41	167,0000	62,7500	79,0000
949	Hep.cel.carcinom+cirrose	56	183	74,75	83,0000
1428	Kronisk autoimmun hepatiti	25	158	56,00	47,0000
922	krypt.cirr.	76	166,00	62,0000	56,0000
378	post.hep.cirr	78	163,0000	59,7500	52,0000
1030		69	165	61,25	70,5000
886	Alc.cir	60,0	165,0	61,25	60
614	Alc.cir	59,0	166,0	62,00	55
208	Alc.cir	56,0	182,0	74,00	62
295	Alc.cir.	53,0	183,0	74,75	84
2442	Alc.cir.	59,0	161,0	58,25	45
239	Alc.cir.	52,0	178,0	71,00	77
101	Alc.cir.	56,0	182,0	74,00	84
2559	Alc.cir.	64,0	190,0	80,00	99
1109	Alc.cir.	65,0	178,0	71,00	92
267	Alc.cir.	50,0	185,0	76,25	100
2394	Alc.cir.	47,0	180,0	72,50	95,5
793	alc.cirr.	42	181,00	73,2500	95,0000
1169	Alc.cirr.	60	180	72,50	76
1989	alc.cirr.	74	162,00	59,0000	68,0000
2629	Alfa-1-antitrypsinmangel	63	179	71,75	95,9
449	alk.cir	45,0	184,0	75,50	101,5
397	alk.cirr	62,0	187,0	77,75	74
623	alk.cirr	45,0	172,0	66,50	90
212	Alk.cirr.	51,0	166,0	62,00	66,4
399	Alk.cirr.	61,0	180,0	72,50	84
300	Alk.cirr.,Hep.C	57,0	169,0	64,25	44,8
1024	Autoimmun hepatitis	48,0	169,0	64,25	50
2449	cir.hep.alc	46	166	62,00	43,0000
1161	cir.hep.alc	61	168	63,50	76,7000
185	cir.hep.alc.o.p.	52	172	66,50	105,0000
1097	Cirr.ex.alc.	61,0	179,0	71,75	113
595	Cirr.ex.alc.	65,0	178,0	71,00	79
2340	Cirr.ex.alc.	48,0	170,0	65,00	62
1961	Cirr.ex.alc.	65,0	169,0	64,25	84

2168	Cirr.ex.alc.	30,0	164,0	60,50	50
3419	Hep. B	47,0	176,0	69,50	78,8
217	Hep.C.	73,0	177,0	70,25	86
2169	Hep.C.	45,0	173,0	67,25	87
2357	Hep.C.	41,0	184,0	75,50	126
2467	Hep.C.	48,0	175,00	68,75	74

BMI	Surface ar	Spironolak	Furosemid	beta-block	Others	ASCITES	COMA	Oesophag
21,2656	1,8300					0	0	0
31,1791	1,98	100+50	80x2	100mg	corodil	0	0	1
22,9854	1,8					0	0	1
26,2188	1,84	200x1	40x1			0	0	1
27,3438	1,73	100x1	40x1			0	0	2
26,5625	1,71	200x1	40x2			0	0	1
24,9649	1,85					0	0	1
28,5185	2,12	100	80x2			0	0	1
25,7228	2	100x2				0	0	2
23,5479	1,99	25x1				0	0	2
20,4529	1,92	100x2	40x2			0	0	2
31,3972	2,25					0	0	1
21,7993	1,73		40x1			0	0	1
31,3469	2,11	100x1				0	0	0
32,4467	1,83	100x1	80x1			0	0	1
23,8437	1,92	50x5				0	0	1
22,2041	1,82					0	0	1
24,4374	1,97	200x1	40x1	80x1		0	0	1
24,4418	1,88					0	0	0
28,1606	1,7200					0	0	3
31,0979	2,0500		40x1			0	0	1
24,5775	1,7	200	120	80		0	0	1
24,2810	1,83	100x1				0	0	1
33,1452	2,33	0	0	0		0	0	1
29,7807	2,1					0	0	1
13,3539	1,73	100		Propranolol R 80		0	0	1
29,9654	1,98					0	0	1
24,9337	1,97					0	0	0
29,4078	2,0000				antabus	0	0	1
25,7117	1,77					0	0	1
29,0286						0	0	3
23,1473	1,63					0	0	1
18,7040	1,52					0	0	0
22,1453	1,74					0	0	0
30,0712	2,04					0	0	0
23,8498	2					0	0	2
25,2493	1,9800	100x1				0	0	0
18,6286	1,6800					0	0	1
26,6097	1,8700	100x1	40x1	inderal 20x3		0	0	1
20,8933	1,5000	25x2				0	0	3
25,6055	1,85					0	0	1
21,2306	1,52	100x1	40x1			0	0	0
16,0156						0	0	1
19,2837	1,5700	100x1		40x1		1	0	1
21,9317	1,7	100	40	40x2		1	0	2
22,3094	1,7800	100x1	80+40			1	0	2
22,3457	1,91	100	40x2			2	0	1
16,4063	1,4000					2	0	1
23,3609	1,7000	200x1	80x2			2	0	2

24,5156	2,06					0	0	2
30,8630	1,88					0	0	
30,5192	2,1					2	0	0
33,0093	2,2100					0	0	0
26,5203	1,7400					0	0	0
33,1398	2,2200	100x1	40x1	80		2	0	2
19,6018		100x1	40x1			0	0	1
22,7204	1,8100	200x1	80+40			2	0	1
29,7521	1,8800	200x2	120x2	80x1		2	0	2
30,0735	2,0800	200x1	80x1	100x1	corodil	2	0	1
12,4213	1,2300	100x1				2	0	
20,0474	1,7200	200+100	80x3		centyl	3	0	0
30,1110	1,9700	50x2	40x2			1	0	1
19,8141	1,5400				eltroxin	0	0	2
26,0933	1,9400	100x1	40x2			1	0	1
28,3266	1,8800			propranolol 40 mg pa		0	0	2
24,7843	2,0500			40x2		0	0	1
18,8271	1,4500			20x1		1	0	2
20,3223	1,6200					0	0	0
19,5717	1,5500	25x2	80x3	sotalol 40x2		1	0	0
25,8953	1,7800					0	0	0
22,0386	1,66					0	0	1
19,9594	1,6		60+30			1	0	2
18,7175	1,81	100x1	40x1			2	0	0
25,0829	2,06	25	0	selozok 50		0	0	3
17,3604	1,44	100				1	0	
24,3025	1,95					1	0	
25,3593	2,05	200	80	80x1		1	0	2
27,4238	2,27					2	0	1
29,0367	2,1	200	160	0		2	0	1
29,2184	2,24	200	80	160		2	1	1
29,4753	2,16	12,5	0	0		2	0	0
28,9979	2,1600	100x2	80x2			2	0	
23,4568	1,95	100	40x2			2	0	1
25,9107	1,7300	100x2	80x1			3	0	1
29,9304	2,15	100x3	120x2		centyl 1x2	3	0	0
29,9799	2,24					3	0	0
21,1616	1,87	100x1				2	0	0
30,4218	2,03					2	0	
24,0964	1,74					0	0	1
25,9259	2,04	100x1				1	0	1
15,6857	1,49					1	0	1
17,5064	1,56					2	0	
15,6046	1,4500	100x2				2	0	0
27,1755	1,8700	50x2	80x2			3	1	2
35,4922	2,1700	100x1	40x2	80x1		3	0	2
35,2673	2,31					0	0	1
24,9337	1,97		20x1			0	0	1
21,4533	1,72	100				2	0	0
29,4107	1,95	100	40x2	Propranolol R 80		3	0	3

18,5901	1,53						3	0	
25,4390	1,95		100		40	Propranolol 40 mg	0	0	1
27,4506	2,03	80x1				40x1	0	0	1
29,0688	2,01	100x1					0	0	1
37,2164	2,46	100x1		80x2			1	0	
24,1633	1,89		200		80		2	0	3

PHG	Hemoglob	Creatinin	Sodium	Potassium	ALAT	Platelets	Bilirubin	Alkaline ph
3	6,7	74	136	4,3000	21,0000		5	138
	6,3	125	131	6,2	33	261	5	190
0	9,9	89	141	4,4	8	277	8	90
1	7,7	124	139	4,9	66	114	19	170
2	6,9	84	144	4,4	26	79	12	112
1	7,8	72	142	4,7	24	84	16	103
1	8,7	60	140		28	90	11	81
1	8,7	95	142	4,1	57		16	229
1	7,4	67	138	5,1	54	205	8	110
0	7,7	71	145	4,5	60	224	9	154
1	8,4	57	140	4,6	13	180	6	114
1	7,3					101		
1	8,5	73	145	3,5	17	144	9	148
0	9,0	97	145	4,7	37	109	14	75
1	5,9	142	128	4,8	51	76	8	174
1	8,9	79	140	4,7	36	89	10	133
1	7,7	75	143	4,6	103	121	9	88
1	6,7	81	136	4,5	36	91	11	181
0	8,7	78	142	4,2	23	169	10	68
	8,0	48	141	4,6	27		10	221
0	9,3	88	144	4,2	19		6	89
0	6,9	87	122	4,6	37	207	18	69
1	10,1	85	139	4,9	21	144	12	105
0	7,7	61	135	4,1	31	129	4	74
0	6,9	61	136	5,3	71	322	13	221
0	8,1	52	132	4,3	45	206	12	103
1	8,7	63	137	3,8	27	100	10	83
0	7,3	144	137	4,1	55	108	11	281
2	8,9	64	141	4,9000	34,0000	410,00	26	275
	8,5	65	142	4,4	104	91	3	116
0	7,7	106	144	4,1	28		3	66
1	8,4	59	140	4,0	59	68	7	209
0	6,4	41	121	4,2	16	244	7	88
3	5,4	88	141	3,2		169		
1	9,4	67	144	4,3	42	86	25	216
1	5,1	63	135	4,2	44	133	13	109
1	7	78	139	4,4	58	492	45	308
1	7,8	102	133	4,8				
3	6,7	159	139	3,9000	19,0000		47	231
2	8	51	142	3,9000	42,0000	377,00	24	326
1	6,7	63	139	3,9	54	65	24	377
0	5,9	90	135	4,5	18	170	26	300
2	7,3	40	142	4,9	36		7	161
3	7,9	52	128	4,5	41,0000		57	1009
2	6	61	136	4,2	23	260	9	94
2	5,4	81	138	3,9	31	261	14	230
1	6,1	74	140	4,6	45	179	10	146
1	6,8							
	6,9	155	133	4,3	28,0000		14	422

2	7,7	73	141	3,7	18	72	20	112
	8,4	66	136	3,5	57	143	27	151
1	10,8	87	143	4,9	60	146	14	112
6	6,2	133	144	4	21		9	74
0	8,2	68	4	141	28		19	162
0	8,5	99	142	4,0	104		10	241
1	7,2	83	138	3,4				
3	7,5	76	139	5,2	85		6	136
1	6,2	95	141	4,3	15		20	246
1	9,1	84	143	3,9	49		19	189
	6,2	97	131	5,3	33		4	140
2	7,4	140	125	4,9	20		17	425
1	7,2	141	142	4,9	60		13	88
2	6,7	69	137	4,1000	39,0000		26	197
0	7,5	94	138	4,2000	30,0000		12	207
	6,9	60	138	4,1000	57,0000	375,00	25	229
	8,1	73	141	3,7	55		13	146
1	6,4	77	140	3,8	57		13	350
0	8,1	112	138	4,3	76	1582	40	263
0	6,5	87	137	4,1	29	575	18	155
1	6,3	68	142	4	20		21	165
0	7,7	35	136	4,4	61	105	40	238
2	6,1	39	138	3,5	25	202	12	127
0	6,7	122	133	3,7	35	97	46	104
1	5,9	125	136	4,1	31	132	218	243
	7,7	48	134	4	35	97	17	148
	8,1	52	139	3,4	27	81	86	162
3	6,8	124	133	4,3	63	647	15	217
1	6,8	54	137	3,9	55	56	54	81
	5,6	53	135	4,1	29	171	19	108
1	6,0	79	135	3,4	17	249	24	155
2	8,5	62	134	3,9	25	106	84	115
	7,5	64	132	3,6000	94,0000	538,00	61	693
1	7,3	100	139	3,5	24		24	114
2	6,4	70	136	4,3000	39,0000	580,00	14	340
0	8,8	83	127	4,0		70		
1	8,9	97	133	5,2	32	149	28	80
1	7,5	94	129	4,7	85	145	32	194
	7,8							
2	6,6	67	135	4,6	52	76	101	118
1	6,0	120	131	4	90	100	56	130
1	7,6	35	137	3,9	136	112	68	332
	6,9	44	137	3,5	30	178	56	285
1	6,4	48	140	3,7	55		57	307
3	4,8	242	141	4,2	74		87	541
2	5,7	124	136	3,3	60		77	335
	8,3	58	137	3,3	53	83	40	83
1	8,3	73	140	3,1	26	90	31	
0	6,5	54	139	3	18	350	109	177
3	7,3	106	136	4,2	37	153	20	224

	6,4	43	136	2,8	15	240	85	174
0	6,3	101	134	4,3	57	70	110	
1	6,5	51	129	4,4	81	51	56	155
0	6,5	111	133	4,3	133	104	83	401
2	6,2	67	133	4,4	67	99	86	145
1	6,7	44	137	3,2	19	88	13	166

Coagulatic	Albumin	Albumin (µ	Biopsy	Child scor	Child scor	Sex	V.hep.cap.	v.hep.free
0,81	42	641	ja	A	4	m	9,50	2,50
0,63	41	621		A	5	m	33,5	11,5
0,87	47	712		A	4	m	11	7
0,54	33,7	511		A	5	m	21,5	12
0,56	41,7	632		A	5	f	25	9
0,6	46	697		A	5	f	20,5	8
0,57	38,2	579		A	5	m	24	7
0,54	45,5	689		A	5	m	20	10
0,57	36,2	548		A	5	m	28,5	10
0,63	43	652		A	5	m	29	9
0,55	40	606		A	5	m	22,5	12,5
0,58				A	5	m	17	14
0,6	45	682		A	5	m	22,5	11,5
0,56	46,9	711		A	5	m	23	6
0,6	42	636		A	5	f	29	13
0,57	43,6	661		A	5	m	24	11
0,59	45	682		A	5	m	22	9
0,52	38	576		A	5	m	31	14
0,61	45	682		A	5	f	13	8
0,78		0	nej	A		f	12,00	7,00
0,85	40,6	615	n	A	4	f	16,00	12,00
0,52	39	591		A	5	f	27	11
0,99	43	652		A	4	m	19	12
0,8	34	515		A	4	m	16	10
0,62	39	591		A	5	m	29	12
0,6	36	545		A	5	m	25	7
0,61	41	621		A	5	m	32	13
0,81	40	606		A	4	m	24,5	10,5
0,56	36	544	ja	A	5	m	20,50	10,00
0,53	42	636		A	5	f	25,6	9
0,59	47,4	718		A	4	m	12	9,5
0,97	37,3	565		A	4	f	24	8,5
0,58	42	636		A	5	m	6	4
0,74		0		A	5	m	21	9,5
0,49	33,1	502		A	5	m	16	7
0,44	29,3	444		B	7	m	25	9
0,37	36,70	556	ja	B	7	m	28,00	9,00
0,50				B	6	m	23,00	5,00
0,52	33	498	ja	B	7	m	25,00	11,00
0,40	39	591	ja	B	6	f	28,50	8,00
0,32	24,9	377		B	8	m	30	11
0,29	26,3	398		B	8	f	24	8
0,65	32,1	486		B	6	f	23,5	11,5
0,79	36,40	552	n	B	8	f	33,50	8,00
0,55	32,3	490		B	8	f	28	9
0,50	34,10	517	ja	B	7	m	19	3,50
0,5	30,2	458	ja	B	8	m	28	9
				B		f	21,00	7,00
0,66		442	ja	B	8	m	28,00	9,00

0,47	37,9	574		B	6	m	23	10
0,45	37,1	562		B	6	m	22	9,5
0,56	35,9	544		B	7	m	14	11
0,43		404	n	B	7	m	23,00	9,50
0,57	26,1	395	n	B	7	f	18,00	10,00
0,74		552	ja	B	7	m	28,00	10,00
0,38	37	555	ja	B	6	f	24,50	8,00
0,65	36,8	558		B	7	m	25,00	6,00
0,57	31	474	ja	B	8	m	33,50	12,00
0,52		568		B	7	m	34,50	6,50
0,60	36,0	545	n	B	7	f	17,00	4,00
0,61	28,6	433	ja	B	8	m	28,00	7,00
0,98	35,3	535		B	6	m	19,00	9,00
0,51	29	439	ja	B	5	f	24,50	5,50
1,30	44	673	ja	B	6	m	36,50	10,50
0,48	34	526	ja	B	6	m	27,00	7,00
0,54		421	nej	B	6	m	27,00	15,00
0,67		633	ja	B	7	f	20,00	8,00
0,70	33,90	514	ja	B	6	f	13,00	6,50
0,52	30,90	468	ja	B	8	f	25,00	7,00
0,52	27,4	415		B	6	f	25,50	11,00
0,49	21	318		C	9	f	36	15
0,33	30	455		C	9	f	24	7
0,46	22	333		C	11	f	28	12
0,4	15	227		C	10	m	34	11
0,44	33	500		C	9	f	22,5	7,5
0,38	25	379		C	12	m	29,5	9
0,4	32	485		C	9	m	34	18
0,36	27	409		C	11	m	33	23
0,43	29	439		C	9	m	29	9
0,34	19	288		C	10	m	32	16
0,29	30	455		C	11	f	36	15
0,31	24	361	ja	C	12	m	32,00	11,00
0,46	28,4	430		C	9	m	34	17,5
0,45	26	392	ja	C	10	m	17,50	9,50
0,52				C		m	31,5	11
0,46	28	424		C	9	m	32	14,5
0,48	25	379		C	11	m	30	12
		0		C		m	26,5	15,5
0,24	29	439		C	9	f	29	11
0,41	23	348		C	12	m	40	17
0,39	30	455		C	11	f	28	5,5
0,47	27	409		C	11	f	30	13
0,41	24,7	374	n	C	12	m	28,50	10,50
0,50	17,9	271	n	C	11	m	46,00	18,00
0,35	21,3	323	ja	C	12	m	27,00	13,00
0,43	29	439		C	11	m	30	15
0,47	32	485		C	9	m	23	11
0,36	20	303		C	12	f	23	10
0,44	23	348		C	10	m	42	21

0,31	16	242	C	12	f	29,5	7,5
0,32	25	379	C	10	m	25	8
0,29	17	258	C	10	m	34	13,5
0,49	20	303	C	10	m	33	11
0,34	20	303	C	12	m	41,5	25
0,45	30	455	C	9	m	29	11

HVPG	PSR	RAP	SYSBT	DIABT	MAP	HR	HBF	ICG-clear
7,00	5,50	1,00	182,00	86,00	123,00	82,00	1,28	545,00
22	15,1	8	135	56	86	69	1,46	499
4	4	5	150	68	98	65	1	430
9,5	5,3	4	121	58	80	53	1,79	101
16	18,8	7	124	64	87	64	0,85	302
12,5	17,9	7	108	56	76	59	0,7	328
17	21	5	155	77	110	79	0,81	281
10	9,7	3	112	66	84	92	1,03	230
18,5	29	6	128	66	89	64	0,637	135
20	8,51	6	149	71	102	94	2,35	330
10	14,3	7	120	70	88	61	0,697	206
3	2	6	166	72	108	67	1,49	422
11	10,3	9	145	79	102	58	1,07	264
17	25,8	5	113	63	81	79	0,657	215
16	11,4	10	156	66	100	79	1,403	251
13	11,1	5	125	65	84	55	1,17	208
13	13	5	145	72	100	50	1,08	498
17	12,1	8	127	67	88	70	1,4	204
5	3	5	123	66	88	72	1,64	846
5,00		5,0000	161	78	111	81		
4,00	205,00	8,0	161	66	103	75	1,61	672,00
16	14,4	8	119	58	81	60	1,1	86
7	8	5	141	92	111	103	0,87	282
6	6,9	7	133	74	99	95	0,86	531
17	9,9	9	125	68	89	74	1,719	182
18	22,9	7	129	88	104	73	0,79	245
19		6	190	95	130	106		
14	7,9	8	141	71	103	62	1,77	380
10,50	29,50	6,00	151,00	80,00	104,00	91,00	0,36	127,00
17,5	20,6	5	172	91	123	67	0,85	301
2,5	1,7	9	172	77	109	43	1,48	757
15,5	12,2	7	158	68	105	63	1,27	479
2	2,7	4	145	70	101	72	0,747	378
11,5	7	6	200	79	127	90	1,64	532
9	18,2	3	145	69	98	80	0,493	182
16	13	7	137	63	90	65	1,23	288
19,00		5,0	120	68	90	86		131,00
18,00	15,60	4,0	184	83	121	64	1,15	128,00
14,00		8,00	163,00	62,00	99,00	54,00		80,00
20,50	17,30	6,00	120,00	63,00	86,00	68,00	1,19	107,00
19	5,5	9	163	86	100	75	3,46	233
16	10,2	4	125	58	87	82	1,56	75
12	11		160	72	101	79	1,086	158
25,50		4,0	111	55	77	77		41,00
19		5	126	54	80	72		152
15,50	3,80	3,0	128	65	83	78		192,00
19	7,2	5	117	71	89	91	2,63	165
14,00	14,00	3,0	125	73	96	73	0,90	251,00
19,00	32,20	4,0	114	64	83	77	0,59	170,00

13	16,7	5	181	78	116	53	0,78	215
12,5	11,3	6	154	69	100	70	1,109	207
3	1,7	8	144	85	106	84	1,76	353
13,50	11,80	6,0000	140	66	93	73	1,14	410,00
8,00	14,00	8,0000	125	63	88	68	0,57	229,00
18,00	13,80	7,0	146	80	106	77	1,30	428,00
16,50	16,50	4,0000	111	53	72	85	0,85	89,00
19,00	18,10	4,0	165	74	109	80	1,05	345,00
21,50	40,90	8,0000	133	53	82	63	0,53	304,00
28,00	11,70	5,0	128	63	89	76	2,40	330,00
13,00	20,30	1,0	92	50	65	87	0,64	222,00
21,00	30,70	3,0	108	57	75	90	0,69	101,00
10,00	9,40	8,0	136	65	91	57	1,06	407,00
19,00	22,10	4,00	170,00	75,00	110,00	70,00	0,86	187,00
26,00	4,60	5,00	134,00	64,00	88,00	80,00	5,64	202,00
20,00	11,40	5,00	139,00	74,00	94,00	98,00	1,76	246,00
12,00	7,50	14,0000	152	72	101	49	1,58	253,00
12,00	12,10	5,0	110	60	79	70	0,99	264,00
6,50	8	5,0	206	87	132	67	0,83	209,00
18,00	20	4,0	161	65	93	75	0,90	124,00
14,50	16,80	9,0000	167	73	109	69	0,86	99,00
21	57,9	3	165	75	104	94	0,363	71
17	19,1	2	158	66	100	86	0,89	96
16	11,9	8	124	64	87	82	1,35	81
23		5	110	53	74	65		83
15	11,1	3	146	71	102	83	1,35	276
20,5		7	151	75	105	64		
16	8,25	6	134	60	86	67	1,94	248
10		5	150	74	99	98		
20	44,4	3	102	54	72	72	0,45	162
16		7	113	78	90	82		
21	9,3	9	116	80	112	103	2,26	79
21,00	13,11	7,00	109,00	61,00	81,00	107,00	1,60	122,00
16,5	24,8	1	137	82	102	83	0,66	106
8,00	7,50	4,00	118,00	57,00	87,00	80,00	1,07	214,00
20,5	10	5	131	66	90	65	2,046	219
17,5		9	122	64	84	71		
18	30,5	11	100	53	73	57	0,59	127
11	10,8	11	116	68	84	79	1,02	370
18	22,8	7	124	58	84	69	0,79	85
23	17	8	132	64	86	105	1,35	114
22,5	37,2	4	146	65	98	81	0,87	63
17		8	112	54	77	69		
18,00		3,0	112	61	81	81		83,00
28,00		5,0	118	56	74	105		95,00
14,00		10,0	111	52	72	67		56,00
15	28,7	9	146	71	98	63	0,523	78
12		7	167	70	108	75		90
13	8,7	3	151	86	110	84	1,5	102
21		10,5	129	64	89	58		89

22		5	121	63	81	69		96
17		4	136	40	85	60		63
20,5		8	147	64	93	70		32
22	19,8	8	119	60	81	80	1,11	78
16,5	18,5	8	138	78	102	91	0,89	68
18	6,29	7	124	70	91	86	2,86	303

GEC	CO-125-I	CI-125-I	CO-99mTc	MT-125-I	MT-99mTc	PV-125-I (L	PV-125-I (r	SVR-125-I
2,36	6,00	3,28	6,00	13,10	13,00	3,75	56,00	1627,00
2,28	8,21	4,15	8,21	9,4	9,4	4,1	46,60	760
2,34	5,42	3,01	5,52	15,5	15,5	4,22	62,10	1372
1,48	3,83	2,08	4,03	18,18	17,92	2,93	39,60	1587
1,42	5,17	2,99	5,17	11,67	11,67	3,4	48,60	1238
1,45	3,89	2,27	3,9	14,8	14,7	3,44	50,50	1419
1,78	6,69	3,62	6,8	12,67	12,19	3,61	49,40	1256
2,25	7,78	3,67	7,78	10,85	10,85	5,33	57,70	833
1,65	5,65	2,83	5,65	18,91	18,28	4,586	55,90	1175
1,7	8,25	4,15	8,5	10,7	10,3	4,55	58,40	930
1,79	4,87	2,54	4,87	13,13	13,13	3,53	50,50	1330
2,95	8,1	3,60	8,1	15,9	15,9	5,5	52,80	1007
1,37	5,96	3,45	6	12,6	12,27	3,41	52,40	1248
2,54	7,3	3,46	7,64	11,61	10,97	3,96	41,20	833
1,43	7,86	4,30	7,86	8,72	8,72	4,36	53,80	916
1,58	6,34	3,30	6,15	16,07	15,63	4,179	55,90	997
2,1	5,84	3,21	5,84	17,8	18,7	5,06	74,40	1301
1,76	5,97	3,03	5,97	14,22	14,22	4,4	56,20	1072
2,23	8,2	4,36	8,3	8,60	8,20	2,89	39,10	810,00
	6,10	3,55	4,50	9,20	9,10	3,37	48,00	1390,00
2,88	7,08	3,45	6,80	16,79	15,50	4,31	46,80	1073,00
1,4	4,91	2,89	4,91	11,13	11,13	3,69	56,80	1189
2,2	6,23	3,40	6,23	22,35	22,35	3,28	46,10	1348
2,52	8,2	3,52	8,2	11,4	11,4	4,49	54,90	898
3,07	6,62	3,15	6,62	12,98	12,98	4,93	53,00	965
1,39	2,92	1,69	2,92	24,34	24,34	3,24	64,80	2658
	9,69	4,89	9,69	9,1	9,1	4	46,70	1024
2,11	5,69	2,89	5,69	18,12	18,12	4,84	61,30	1335
1,65	9,90	4,95	9,40	10,90	10,50	4,42	50,90	800,00
1,74	3,56	2,01	3,56	12,3	12,3	2,76	39,40	2651
2,75	5,34	#DIV/0!	5,34	18,98	18,98	4,36	49,10	1498,00
1,97	3,99	2,45	4,17	13,41	12,60	2,67	44,40	1965,00
1,85	4,78	3,14	4,78	15,6	15,6	3,38	67,60	1623
2,09	6,37	3,66		15,6	15,6	4,22	65,90	1520
1,4	8,2	4,02	7,9	9,16	8,59	3,62	40,20	927
1,53	8,25	4,13	8,47	13,66	13,16	5,21	65,90	805,00
1,51	8,50	4,29	8,20	8,80	8,70	4,69	58,70	800,00
1,13	5,90	3,51	5,70	15,40	14,10	3,82	67,80	1586,00
1,13	10,50	5,61	8,70	10,21	10,23	4,33	56,90	693,00
1,29	7,98	5,32	7,59	9,53	8,99	3,04	59,00	802,00
1,54	8,89	4,81	9,22	10,23	10,09	3,99	53,90	819,00
0,87	7,52	4,95	7,78	9,84	9,36	3,10	58,40	882,00
1,13		#DIV/0!						
1,27	7,33	4,67	7,27	7,93	7,51	3,26	62,10	796,00
1,38	6,75	3,97	8,89	10,23	10,15	3,53	57,00	889
1,38	4,90	2,75	4,60	16,40	16,50	3,70	56,00	1306,00
1,52	9,22	4,83	9,57	11,17	10,93	4,67	64,50	728
1,00	4,50	3,21	4,50	13,90	14,20	3,07	72,30	1653,00
1,50	4,82	2,84	4,62	11,08	10,23	3,59	56,40	1308,00

1,74	7,13	3,46	7,13	18,4	18,4	5,27	63,50	1245
1,71	6,9	3,67	6,9	10,3	10,3	4,15	50,60	1090
1,92	8,75	4,17	8,68	11,66	11,76	5,00	53,20	896,00
2,11	10,50	4,75	10,73	9,56	9,52	5,28	50,80	663,00
1,09	5,21	2,99		15,97	15,90	3,22	51,90	1228,00
2,14	6,81	3,07	6,64	11,50	10,80	3,93	37,40	1663,00
		#DIV/0!						
1,60	7,30	4,03	7,19	11,19	10,73	3,71	54,60	1150,00
1,53	6,00	3,19	6,30	16,10	16,30	4,86	59,90	987,00
1,84	8,61	4,14	8,19	10,65	10,17	4,06	44,10	780,00
0,67	4,08	3,32	3,94	12,00	11,54	2,44	77,20	1255,00
1,19	6,39	3,72	6,25	11,35	11,27	3,24	53,90	901,00
1,30	7,95	4,04	7,84	13,66	13,34	3,99	46,40	835,00
1,39	4,91	3,19	4,44	16,40	16,20	3,50	67,30	1727,00
1,91	11,28	5,81	8,33	11,23	10,41	4,68	59,30	589,00
2,13	13,40	7,13	10,90	7,50	7,27	5,44	68,90	531,00
1,70	7,14	3,48	7,01	14,89	14,46	4,49	54,10	975,00
1,34	4,18	2,88	4,00	12,64	12,18	2,73	58,10	1416,00
1,46	4,56	2,81	4,33	16,21	15,83	2,83	50,40	2228,00
	4,29	2,77	4,05	16,35	16,92	2,57	49,50	1660,00
1,58	7,49	4,21		11,92	11,74	3,96	56,10	1068,00
1,39	9,5	5,72	9,5	11,76	11,76	4,62	77,20	849
1,1	8,03	5,02	7,47	9,7	9,42	3,32	60,40	976
1,26	6,04	3,34	5,93	12,25	11,75	3,97	64,10	1046
1,68	9,81	4,76	9,1	14,8	13,9	6,11	72,70	563
1,2	4,34	3,01	4,49	15,7	15,4	2,63	58,50	1825
	7,51	3,85	7,43	12,06	11,97	4,37	56,80	1044
2,13	6,39	3,12	6,79	17,05	15,13	4,76	56,70	1002
	9,66	4,26	9,66	13,56	13,56	5,6	56,80	745
1,45	9,4	4,48	9,49	14,6	14,7	5,85	63,60	587
	8,69	3,88	8,64	12,84	12,84	5,15	51,30	764
1,75	13,52	6,26	13,66	8,46	8,19	5,6	58,40	609
1,22	14,40	6,67	14,20	6,08	5,99	5,38	56,60	411,00
0,66	6,69	3,43		13,97		4,41	58,00	1208
1,74	5,58	3,23	5,23	19,30	19,10	4,37	64,30	1190,00
1,87	11,88	5,53	8,8	16,11	16,00	7,47	77,90	572,00
1,76	7,39	3,30	7,39	12,2	12,2	5,11	50,30	812
1,4	5,69	3,04	5,69	14,2	14,2	3,58	48,40	871
2,01	5,71	2,81	5,71	11,06	11,06	3,82	42,50	1023
1,04	8	4,60	8,2	10	10,1	4,42	66,60	770
1,81	9,01	4,42	9,01	9,76	9,76	4,75	56,60	693
0,86	6,82	4,58	6,82	7,78	7,78	2,96	65,80	1103
1,04	7,8	5,00	7,8	11,12	11,12	3,8	75,90	708
0,87	6,27	4,32	6,69	10,50	10,30	3,09	71,90	995,00
1,44	8,10	4,33	7,89	11,49	11,09	3,75	48,70	681,00
1,85	9,42	4,34	9,57	12,58	11,87	5,63	53,60	696,00
1,72	7,15	3,10	7,15	14,87	14,87	4,68	41,40	996
1,82	7,92	4,02	7,92	9	9	3,86	48,90	1020
1,38	4,38	2,55	5,57	13,5	12,5	3,53	57,00	1954
1,65	4,504	2,31	4,91	16,15	15,34	3,49	41,50	1396

1,02	5,63	3,68	5,63	13,13	13,13	3,16	63,20	1080
1,2	7,53	3,86	7,53	10,7	10,7	4,3	54,40	861
1,4	7	3,45	7,39	18,68	18,08	4,19	48,70	971
1,38	8,34	4,15	8,34	10,67	10,67	4,19	48,20	700
2,24	10,25	4,17	10,25	7,3	7,3	5,12	40,60	734
2,32	5,04	2,67	5,04	18,38	18,38	4	54,20	1333

CBV (L)	CBV (ml/k	CBV (ml/k	Total lean	Total fat m	Total BMC	Total DEX	SV	PP
1,30	19,40	18,30	51416,00	12784,00				
1,28	14,55	20,16	53706,00	31534,00	2528,00	87768,00	118,9855	79
1,4	20,59	21,05	52528,00	10179,00	2701,00	65408,00	83,38462	82
1,14	15,41	17,95	55272,00	15714,00	3543,00	74529,00	72,26415	63
1,01	14,43	17,57	36286,00	31000,00	2129,00	69415,00	80,78125	60
0,95	13,97	16,52	34087,00	32479,00	2133,00	68699,00	65,9322	52
1,36	18,63	20,68	50393,00	19982,00	2703,00	73078,00	84,68354	78
1,41	15,26	19,45	65287,00	22973,00	3630,00	91890,00	84,56522	46
1,72	21,10	24,23	53499,00	24826,00	2888,00	81213,00	88,28125	62
1,46	18,72	19,73				0,00	87,76596	78
1,07	15,29	14,03				0,00	79,83607	50
2,15	20,67	29,05	71186,00	29005,00	3632,00	103823,00	120,8955	94
1,22	19,37	18,77	47548,00	13392,00	2594,00	63534,00	102,7586	66
1,33	13,85	19,35	61078,00	31431,00	3362,00	95871,00	92,40506	50
1,14	14,07	20,36	40391,00	37801,00	2458,00	80650,00	99,49367	90
1,65	22,09	23,49	57864,00	13543,00	3344,00	74751,00	115,2727	60
1,73	25,44	25,16	51273,00	14042,00	3033,00	68348,00	116,8	73
1,41	18,01	19,65	50121,00	25061,00	3046,00	78228,00	85,28571	60
1,12	15,14	16,47	43340,00	27275,00	3151,00	73766,00	113,8889	57
0,93	13,16	16,52	40590,00	27109,00				
1,83	19,88	27,52						
0,91	14,02	15,33	36325,00	25111,00	2427,00	63863,00	81,83333	61
2,32	32,68	35,29	45177,00	23961,00	2412,00	71550,00	60,48544	49
1,55	13,96	20,74	74459,00	33497,00	2865,00	110821,00	86,31579	59
1,43	15,33	20,36	57540,00	32383,00	3387,00	93310,00	89,45946	57
1,18	23,60	14,28	37947,00	7980,00	2598,00	48525,00	40	41
1,47	16,97	22,62					91,41509	95
1,72	21,77	24,23	55314,00	21727,00	2530,00	79571,00	91,77419	70
1,73	19,89	26,02	54587,00	29260,00				
0,72	10,29	11,76	41389,00	25617,00	2656,00	69662,00	53,13433	81
1,68	18,90	24,44	64838,00	20580,00	3465,00	88883,00	124,186	95
0,84	14,00	14,42	32367,00	24787,00	2299,00	59453,00	63,33333	90
1,24	24,80	20,62	38539,00	8772,00	2734,00	50045,00	66,38889	75
1,66	25,9375	25,53846	48949	12222	3341	64512,00	70,77778	121
1,17	13	17,39777	58572	28404	3131	90107,00	102,5	76
1,81	22,91	24,46	57314,00	18601,00	3310,00	79225,00	126,9231	74
1,26	15,76	17,75	48578,00	28250,00				
1,39	24,58	20,44	43276,00	10659,00				
1,79	23,56	27,84	46799,00	25958,00				
1,20	23,22	21,70	36607,00	12876,00				
1,50	20,27	23,08	56222,00	16159,00	2495,00	74876,00	118,5333	77
1,17	22,08	20,89	30955,00	19518,00	2376,00	52849,00	91,70732	67
	0,00	0,00	26848,00	11964,00	1746,00	40558,00	0	88
0,92	17,47	14,97	29750,00	20487,00				
1,14	18,4168	17,95276	40111	19332	2288	61731,00	93,75	72
1,35	20,41	20,26	43361,00	20108,00				
1,68	23,20442	23,17241	52777	16731	3075	72583,00	101,3187	46
0,93	21,93	16,17	35127,00	5094,00				
0,82	12,92	13,38	46023,00	15016,00				

2,18	26,27	28,87	57929,00	21086,00	3363,00	82378,00	134,5283	103
1,18	14,39	19,75	50704,00	28751,00	2476,00	81931,00	98,57143	85
1,70	18,09	24,59	65598,00	25033,00	3079,00	93710,00	104,1667	59
1,67	16,02	23,64602	67529,00	33098,00	3274,00	103901,00		
1,38	19,84	23,38983	35256,00	31900,00	2263,00	69419,00		
1,23	11,67	17,32394	71209,00	31122,00	2823,00	105154,00		
			32110,00	28140,00				
1,30	19,14	19,32	47476,00	17936,00	2640,00	68052,00		
1,63	20,12	26,59	56164,00	21890,00				
1,46	15,85	21,23636	57651,00	31212,00	3291,00	92154,00		
0,78	24,83	13,66						
1,20	20,00	17,80	49874,00	7376,00				
1,77	20,55	27,54864	49321,00	33622,00	2837,00	85780,00		
1,32	25,49	22,37	34529,00	15662,00				
1,96	24,81	28,82						
1,62	20,51	25,80	52992,00	22889,00				
1,72	20,72	23,01	60636,00	19305,00				
0,85	18,05	15,17857	28883,00	16510,00	2118,00	47511,00		
1,20	21,48	19,40						
1,21	23,27	20,24	36848,00	13768,00				
1,47	20,79	24	40067,00	29008,00	2099,00	71174,00		
1,86	31,00	30,37					101,0638	90
1,26	22,91	20,32					93,37209	92
1,18	19,03	15,95					73,65854	60
2,27	27,02	30,37	63586,00	16859,00	3745,00	84190,00	150,9231	57
1,11	24,67	19,06					52,28916	75
1,12	14,55	15,77					117,3438	76
1,61	19,17	21,76	58000,00	22800,00	3400,00	84200,00	95,37313	74
2,1	21,21	26,25					98,57143	76
2,29	24,89	32,25	60700,00	28700,00	2880,00	92280,00	130,5556	48
1,86	18,60	24,39					105,9756	35
1,84	19,27	25,38					131,2621	36
1,44	15,10	19,66	67909,00	24643,00				
1,56	20,52632	21,51724	56029	16830	3964	76823,00	80,60241	55
1,78	26,12	30,17	42846,00	22888,00				
2,35	24,50	32,75	58117,00	32516,00	3768,00	94401,00	182,7692	65
1,5	14,78	19,87	75065,00	22751,00	4017,00	101833,00	104,0845	58
1,35	18,24	17,36	59229,00	12581,00	3218,00	75028,00	99,82456	47
1,05	11,67	15,79	53590,00	33965,00	2473,00	90028,00	72,27848	48
1,35	20,33	21,77	38867,00	24911,00	2794,00	66572,00	115,942	66
1,47	17,50	20,28	65221,00	16008,00	3040,00	84269,00	85,80952	68
0,89	19,87	13,85	30807,00	12028,00	2010,00	44845,00	84,19753	81
1,45	29,00	22,57	35988,00	11880,00	2329,00	50197,00	113,0435	58
1,08	25,03	17,42	34114,00	7192,00				
1,50	19,52	23,58						
1,86	17,75	28,00	69809,00	30921,00				
1,77	15,66	24,67	66775,00	42622,00	3771,00	113168,00	113,4921	75
1,19	15,06	16,76	51950,00	24658,00	2999,00	79607,00	105,6	97
0,91	14,68	14,00	47531,00	11898,00	2739,00	62168,00	52,14286	65
1,15	13,69	17,90	68874,00	11626,00	3429,00	83929,00	77,65517	65

1,23	24,60	20,33					81,5942	58
1,34	17,01	19,28	54739,00	20963,00	3140,00	78842,00	125,5	96
2,11	24,53	30,04					100	83
1,48	17,01	22,01					104,25	59
1,25	9,92	16,56					112,6374	60
1,54	20,81	22,40					58,60465	54

AC	Htc	Blodvolum	Non-CBV	pH	PaCO2	PaO2	St.HCO3	O2SAT
1,51	0,22	5,10	3,82	7,39	4,6	12,10	21,40	97,40
1,02	0,41	6,64	5,24	7,38	5,2	9,20	23,00	93,80
1,15	0,36	4,31	3,17	7,43	4,7	12,50	24,30	97,00
1,35	0,25	4,37	3,36	7,36	4,4	11,20	19,50	96,00
1,27	0,24	4,37	3,42	7,43	4,47	12,60	23,00	97,80
1,09	0,39	5,53	4,17	7,41	4,8	11,20	23,30	97,00
1,84	0,38	8,05	6,64	7,44	4,4	14,20	23,50	98,00
1,42	0,34	6,58	4,86	7,40	4,6	10,60	22,10	95,00
1,13	0,35	6,61	5,15	7,43	4,7	9,20	23,50	94,00
1,60	0,36	5,19	4,12	7,37	4,6	13,40	20,80	97,00
1,29	0,32	7,69	5,54	7,43	4,8	12,10	24,30	97,00
1,56	0,37	5,08	3,86	7,42	5,1	14,10	25,10	98,00
1,85	0,38	5,98	4,65	7,42	5	10,00	24,40	95,00
1,11	0,24	5,54	4,40	7,38	4,3	9,50	19,80	94,00
1,92	0,42	6,67	5,02	7,40	4,9	10,30	23,00	95,00
1,60	0,31	6,99	5,26	7,37	6	12,40	24,40	95,00
1,42	0,31	6,08	4,67	7,40	4,1	14,40	20,70	97,00
2,00	0,36	4,25	3,13	7,42	5,3	14,00	25,60	97,00
1,34	0,26	4,80	3,89	7,44	4,8	11,70	24,90	97,00
1,23	0,40	5,09	2,77	7,40	4	12,00	20,60	97,00
1,46	0,34	6,44	4,89	7,42	4,5	13,10	22,70	97,00
1,57	0,30	6,73	5,30	7,36	4,4	11,80	19,40	96,00
0,98	0,38	4,90	3,72	7,43	4,4	13,10	23,30	97,00
0,96	0,38	6,04	4,57	7,42	5,5	11,60	26,10	97,00
1,31	0,31	6,68	4,96	7,42	4,5	12,60	22,90	97,00
0,66	0,36	4,06	3,34	7,41	5,4	12,30	25,40	98,00
1,31	0,33	6,17	4,49	7,39	5,19	11,50	22,80	98,00
0,70	0,31	3,69	2,85	7,40	5,7	10,30	23,30	96,00
0,89	0,28	4,50	3,26	7,42	4,2	15,40	21,30	97,00
0,58	0,25	5,43	3,77	7,37	6,7	17,30	27,30	96,00
1,35	0,40	5,62	4,45	7,40	5,1	9,70	23,50	96,00
1,72	0,22	6,48	4,67	7,45	4,2	11,10	23,10	97,00
1,54	0,26	5,19	3,69	7,45	4,5	12,60	24,30	98,00
1,37	0,29	4,17	3,00	7,44	4,6	11,80	24,40	100,00
0,00		0,00	0,00					
1,30	0,28	4,70	3,56	7,43	5,6	10,20	26,90	96,00
2,20	0,28	6,22	4,54	7,44	4,1	13,10	22,00	97,00

1,31	0,36	7,75	5,57	7,40	4,71	11,82	24,60	98,00
1,16	0,35	6,03	4,85	7,42	6,1	7,50	28,30	91,00
1,77	0,46	8,47	6,77	7,42	5,4	8,90	25,50	94,00
1,12	0,32	6,46	4,60	7,40	4,99	10,30	24,80	97,00
1,01	0,29	4,47	3,21	7,40	4,7	11,00	22,40	98,00
1,23	0,31	5,48	4,30	7,46	4,30	10,70	24,60	98,00
2,65	0,27	8,04	5,77	7,44	3,5	10,20	20,10	96,00
0,70	0,32	3,68	2,57	7,43	4,5	12,00	23,00	98,00
1,54	0,32	6,11	4,99	7,40	5,2	11,20	24,20	99,00
1,29	0,30	6,49	4,88	7,47	4,12	9,70	24,10	96,00
1,30	0,28	7,46	5,36	7,44	4,8	10,40	25,00	98,00
2,72	0,23	7,36	5,07	7,45	4	11,20	22,20	97,00
3,03	0,30	7,03	5,17					
3,65	0,34	8,03	6,19	7,46	4,3	8,40	24,00	96,00
1,47	0,30	6,02	4,46	7,45	4,4	12,20	24,00	97,00
2,81	0,33	10,58	8,23	7,47	4,1	11,10	22,80	96,00
1,79	0,34	7,33	5,83		4,5	11,80		96,00
2,12	0,32	5,01	3,66	7,45	4,3	10,70	23,30	96,00
1,51	0,34	5,48	4,43	7,42	6,4	8,70	28,90	93,00
1,76	0,27	5,82	4,47	7,42	4,9	10,20	23,80	96,00
1,26	0,23	5,97	4,50	7,50	4,1	9,50	25,40	95,00
1,04	0,34	4,24	3,35	7,43	5,4	11,10	26,40	96,00
1,95	0,32	5,31	3,86	7,45	4,1	9,40	22,70	94,00
1,51	0,35	6,80	5,03	7,46	4,8	10,40	26,30	96,00
1,09	0,32	5,40	4,21	7,45	4,1	11,20	22,60	96,00
0,80	0,27	4,65	3,74	7,46	3,93	10,80	24,20	96,00
1,19	0,31	4,82	3,67	7,46	4,3	10,30	24,00	94,00

1,41	0,31	4,36	3,13	7,52	4,9	8,80	30,40	94,00
1,31	0,24	5,47	4,13	7,44	4	13,10	22,20	97,00
1,20	0,28	5,58	3,47	7,41	5,2	8,80	24,30	96,00
1,77	0,29	5,65	4,17	7,40	5,3	11,50	24,50	98,00
1,88	0,27	6,74	5,49	7,42	4,7	14,30	23,60	99,00
1,09	0,33	5,66	4,12	7,46	4,6	10,30	25,00	98,00

Hb	AaPO2
	2,22
	4,4
	1,7
	3,36
	1,876
	2,88
	0,36
	3,72
	5
	0,92
	1,98
	-0,38
	3,84
	5,18
	3,66
	0,24
	0,52
	-0,52
	2,38
	3,04
	1,34
	2,76
	1,46
	1,64
	1,84
	1,06
	2,112
	2,7
	-0,6
	-5,5
	4,02
	3,7
	1,84
	2,52
	19,84
	2,92
	1,82

2,368
5,02
4,46
3,552
3,2
3,98
5,44
2,44
2,4
5,196
3,68
3,84
19,84
6,28
2,36
3,82
2,64
3,98
3,46
3,76
5,42
2,26
5,52
3,68
3,72
4,324
4,38

5,16
1,94
4,8
1,98
-0,1
4,02
